# A Dynamic Access Probability Adjustment Strategy for Coded Random Access Schemes

**DOI:** 10.3390/s19194206

**Published:** 2019-09-27

**Authors:** Jingyun Sun, Rongke Liu, Enrico Paolini

**Affiliations:** 1 School of Electronics and Information Engineering, Beihang University, 37 Xueyuan Road, Haidian District, Beijing 100191, China; sunjingyun@buaa.edu.cn; 2 Department of Electrical, Electronic, and Information Engineering, University of Bologna, via Dell’Universitá 50, 47522 Cesena (FC), Italy

**Keywords:** congestion, estimation, irregular repetition slotted ALOHA, medium access control, random access, successive interference cancellation

## Abstract

In this paper, a dynamic access probability adjustment strategy for coded random access schemes based on successive interference cancellation (SIC) is proposed. The developed protocol consists of judiciously tuning the access probability, therefore controlling the number of transmitting users, in order to resolve medium access control (MAC) layer congestion states in high load conditions. The protocol is comprised of two steps: Estimation of the number of transmitting users during the current MAC frame and adjustment of the access probability to the subsequent MAC frame, based on the performed estimation. The estimation algorithm exploits a posteriori information, i.e., available information at the end of the SIC process, in particular it relies on both the frame configuration (residual number of collision slots) and the recovered users configuration (vector of recovered users) to effectively reduce *mean-square error* (MSE). During the access probability adjustment phase, a target load threshold is employed, tailored to the packet loss rate in the finite frame length case. Simulation results revealed that the developed estimator was able to achieve remarkable performance owing to the information gathered from the SIC procedure. It also illustrated how the proposed dynamic access probability strategy can resolve congestion states efficiently.

## 1. Introduction

In machine-type and Internet-of-Things (IoT) communications, users generate a large amount of bursty traffic to transmit over a shared communication medium. Coordinated multiple access schemes turn to be impractical and generally inefficient in such scenarios. For this reason, random access schemes have attracted a renewed interest, as they provide a practical way for uncoordinated users to contend for channel resource.

Pure ALOHA scheme [1] was proposed in 1968 to share a channel among a number of users sending packets as soon as they have data to transmit. Classical slotted ALOHA [2] is a distributed random access scheme in which time is divided into slots of equal duration with each transmission starting only at the beginning of a time slot. In both variants, an absence of coordination among users may lead to collisions (two or more packets are received in overlapping time windows). All packets involved in a collision are often reported as useless and are retransmitted after a random delay, according to some probability distribution, or (in the framed case) in the next frame. As a result, pure ALOHA and slotted ALOHA suffer from a throughput penalty and an under-utilization of channel resource. The optimal normalized throughput of pure ALOHA is 0.18 and the throughput of slotted ALOHA is increased to 0.37.

The expression of “coded random access” refers to a set of random access schemes that combine the packet repetition of users with successive interference cancellation (SIC) at the receiver. The first coded random access scheme is collision resolution diversity slotted ALOHA (CRDSA) [3], where each user sends two packet replicas in two random slots of the frame, and then SIC is applied to recover the collided packets in an iterative fashion. After CRDSA, CRDSA++ [4] was proposed to further improve throughput by increasing the number of packet replicas. In [5], where irregular repetition slotted ALOHA (IRSA) was proposed, the SIC-based random access process is conveniently described by a bipartite graph, establishing a bridge between the SIC procedure and the iterative erasure decoding of graph-based codes. In IRSA, the packet repetition rate is irregular from user to user and is chosen independently by each active user according to a suitably designed probability distribution. Since then, coded random access emerged as a new paradigm and has been the subject of several investigations over the past few years (e.g., [6,7,8,9,10,11,12] and references therein). As a result, the throughput has substantially increased which makes it a practical and efficient solution to support uncoordinated access.

Despite their numerous advantages, coded random access schemes exhibit lower critical points in traffic load. In other words, the throughput of these schemes is maximized for load values less than 1 and, for larger values of the load, it decreases very rapidly. Congestion occurs when the number of active users is greater than the receiver processing capacity. Several control methods for random access schemes have been investigated, which may be classified into two kinds: Dynamic frame length based methods and dynamic access probability based methods. In dynamic framed slotted ALOHA (DFSA) systems, the frame size is adjusted dynamically according to the estimated number of active users in order to maximize the system efficiency [13,14,15,16,17]. In dynamic access probability based schemes, on the other hand, an access controller is required to adjust the users access probability under high traffic loads in order to limit the number of transmitting users [18,19,20,21]. However, in [18,19,20], the estimation process was simply based on the status of frame slots before the application of SIC and in [21], the estimation is assumed to be ideal at the receiver. Furthermore, the proposed random access control mechanisms in [18] are based on random access schemes without SIC at the receiver, which is not applicable for coded random access schemes. In both [19,20], users directly employ the load threshold from [5], which is obtained via asymptotic analysis (frame length and user population size tending to infinity, their ratio remaining constant). When applied to the finite frame length case, asymptotic load thresholds tend to be beyond the actual critical point, which may yield considerable throughput losses.

In this paper, a dynamic access probability based strategy for coded random access schemes is proposed to resolve congestion. The proposed strategy performs two main tasks: Estimation of number of transmitting users in the current frame and the adjustment of access probability in the next frame based on the estimation results. In our previous work [22,23,24] techniques for a more reliable estimation of the number of transmitting users in coded random access schemes were developed and more specially, the number of transmitting users in the current frame was estimated using a posteriori information gathered throughout the SIC process. A posteriori estimation was considered for CRDSA in [22], for IRSA in [23] and for CRDSA over a packet and slot erasure channel in [24]. Notably, [22,23,24] were entirely focused on the estimation process, without any attempt to exploit it within a dynamic access probability adjustment protocol. The usage of a target load threshold tailored to the finite frame length case and the introduction of a state judgment to avoid not fully reliable estimation in high traffic load conditions are other original features of this manuscript.

The system model and some preliminary definitions are provided in Section 2. The estimation algorithm for the number of transmitting users in the current frame is addressed in Section 3.1, while the access probability adjustment strategy is proposed in Section 3.2. Numerical results are illustrated in Section 4 and concluding remarks are given in Section 5.

## 2. Preliminaries

### 2.1. System Model

We consider a scenario where multiple users contend for access to a single central receiver. The medium access control (MAC) layer is organized into frames and the random access scheme is a slotted one. We denote random variables by capital letters, while their realizations and deterministic quantities are denoted by lower case letters. The frame length is fixed and divided into *m* time slots with equal duration.

Active users are the ones who have packets to transmit. Congestion occurs when the number of active users is too large in comparison to the available resources (a more precise definition of congestion is given in Section 3.2). We use the subscript (k) to represent the index of the MAC frame. If there is no congestion or the congestion is resolved, the index (k) is re-initialized to (0) in the next frame, otherwise, it keeps counting. As such, a frame index k≥1 indicates that we are in the *k*-th frame of the current congestion event.

User population size is $n_{\mathrm{pop}}$. The number of active users is unknown to the receiver and is modeled by a random variable $N_{a}$; the number of active users at the beginning of frame *k* is $N_{a}^{\left(k\right)}$. No new user activates before the current congestion has been resolved. Denoting by $\Delta^{\left(k\right)}$, the number of users that are recovered while processing frame *k*, for k≥1 we have:

(1) $$\begin{array}{c} N_{a}^{\left(k\right)}=N_{a}^{\left(k-1\right)}-\Delta^{\left(k-1\right)}. \end{array}$$

Transmitting users are the ones who are allowed to transmit their packets in the frame. Let $T_{a}^{\left(k\right)}$ be the number of transmitting users during frame *k*. Moreover, denote by $p_{ac}^{\left(k\right)}$ the access probability of the active users during frame *k*. At the beginning of the *k*-th frame, each active user becomes a transmitting one with probability $p_{ac}^{\left(k\right)}$, independently of other active users. Hence, the conditional expected value of $T_{a}^{\left(k\right)}$ is:

(2) $$\begin{array}{c} E\left[T_{a}^{\left(k\right)}|n_{a}^{\left(k\right)}\right]=n_{a}^{\left(k\right)}p_{ac}^{\left(k\right)}. \end{array}$$

Each transmitting user is frame- and slot- synchronous and attempts at most one packet transmission per frame.

In every frame corresponding to k=0, all active users transmit their packets to the receiver, i.e., we have $p_{ac}^{\left(0\right)}=1$ and $t_{a}^{\left(0\right)}=n_{a}^{\left(0\right)}$. The instantaneous channel load over frame *k* is defined as:

(3) $$G^{\left(k\right)}=\frac{t_{a}^{\left(k\right)}}{m}$$

and represents the average number of packet transmissions per slot. The throughput over frame *k* is defined as:

(4) $$T_{h}^{\left(k\right)}=\frac{t_{a}^{\left(k\right)}}{m}\left(1-P_{L}\right)$$

representing the average number of successfully recovered packets per slot by the receiver. The quantity $P_{L}$ in Equation (4) is the packet loss rate over the frame, which is expressed as:

(5) $$P_{L}=1-\frac{\delta^{\left(k\right)}}{t_{a}^{\left(k\right)}}.$$

In IRSA, each transmitting user sends *L* packet replicas to slots picked uniformly at random. The number of replicas, named user degree, is a discrete random variable probability mass function (p.m.f.) $\left\{\Lambda_{l}\right\}$, where $\Lambda_{l}=P\left(L=l\right)$ is the probability that a user generates *l* packet replicas. Users choose their replica factor (i.e., user degree) *L* independently of each other, with no coordination, and the values of user degree are according to distribution $\left\{\Lambda_{l}\right\}$. We also represent $\left\{\Lambda_{l}\right\}$ in polynomial form, as $\Lambda \left(x\right)=\sum_{l=1}^{d_{\max}}\Lambda_{l}x^{l}$, where $d_{\max}$ is the maximum number of packet replicas per user. Both information about the transmitting user index (assuming users are indexed from 1 to $n_{\mathrm{pop}}$) and pointers to the slots where the other replicas have been transmitted are included in the header of each packet replica. CRDSA can be seen as IRSA with $\Lambda \left(x\right)=x^{d_{\max}}$.

In this paper, a classical collision channel model is adopted. After packet replica transmissions, each slot takes one of the following three states: Empty slot (no packet replica transmitted in that slot), singleton slot (only one packet replica transmitted in that slot), and collision slot (two or more than two packet replicas transmitted in that slot). The receiver can always correctly classify the state of each slot. Collision slots provide no information to the receiver about the number and content of collided packet replicas directly. However, as soon as the contribution of interference, generated by some transmitting users on the slot, is canceled and only one packet replica is left in it, the slot status is updated to singleton. Similarly, if all of the packet replicas transmitted in the slot are recovered by the receiver, the slot status (singleton slot or collision) is updated to empty. Packet replicas from singleton slots are always correctly received, which means that packet losses may only be generated by unresolved collisions.

After transmissions, the pointers to twin replicas in the header of the packet enable SIC at the receiver. At first, the receiver stores the content of the frame. Then, the receiver performs iteratively the following procedure, consisting of two subsequent steps:

1. Pick out the singleton slots in the frame. For each singleton slot, extract the transmitting user index, the content of the packet replica, and positions of other twin replicas. Identified users in this step become recovered users;

2. For each user recovered at step 1, remove the user’s contribution of interference in the slots where the packet replicas have been transmitted. A new singleton slot will appear if, after interference cancellation, they contain only one replica.

The iterative SIC procedure terminates when all slots are empty ones, in which case SIC succeeds, or when no singleton slot can be found but collision slots still exist, in which case it fails. At the end of the SIC procedure, the residual number of empty slots in the frame is denoted by $M_{e}$, and the residual number of collided slots per frame by $M_{c}$. Obviously, we have $M_{e}+M_{c}=m$.

**Figure 1:**
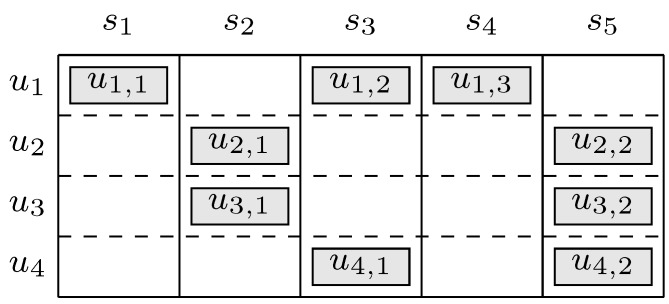
Example of a MAC frame with $t_{a}=4$ transmitting users and m=5 slots. User $u_{1}$ sends three packet replicas and the other users each send two packet replicas. Slots $s_{1}$ and $s_{4}$ are singleton slots and the left $s_{2}$, $s_{3}$, and $s_{5}$ are collision slots.

**Example** **1.** *With reference to Figure 1, $t_{a}=4$ users transmit their packets to a frame with m=5 slots. User $u_{1}$ generates three replicas of his packet, and sends them to $s_{1}$, $s_{3}$, and $s_{4}$, respectively. Each of the other users generate two replicas of the corresponding packets and transmit them as illustrated in the figure. At the receiver, slots $s_{1}$ and $s_{4}$ are singleton slots and the left $s_{2}$, $s_{3}$, and $s_{5}$ are collison slots.*

**Figure 2:**
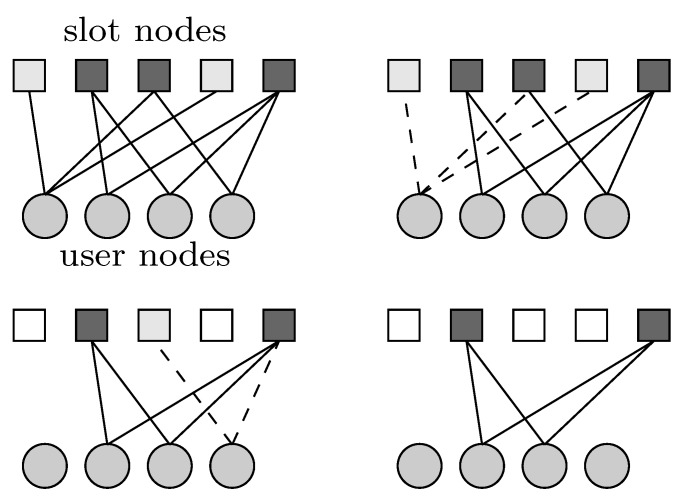
Example of successive interferece cancellation (SIC) procedure corresponding to Figure 1. Squares correspond to slots and circles correspond to users.

*Figure 2 provides a graphical interpretation (first proposed in [5]) of the iterative SIC procedure performed on the frame of Figure 1. In the presented graph, “slot nodes" represent slots and “user nodes" represent users. In the first SIC iteration, $s_{1}$ and $s_{4}$ are singleton slots and the corresponding packet replicas are correctly received, making $u_{1}$ a recovered user. The pointer to slot $s_{3}$, where the twin of the replica in $s_{1}$ has been transmitted, is extracted (step 1). After the interference from recovered user $u_{1}$ in slot $s_{3}$ is canceled and only one packet replica is left in $s_{3}$, making $s_{3}$ a new singleton slot (step 2). Then a second iteration is triggered. After three SIC iterations, users $u_{2}$ and $u_{3}$ remain unrecovered, there are no singleton slots in the frame, and SIC terminates with failure.*

*The feedback frame configuration signal is {0,1,0,0,1} which indicates that $s_{1}$, $s_{3}$ and $s_{4}$ are empty slots and that $s_{2}$ and $s_{5}$ are unresolved collision slots. Receiving this feedback signal, $u_{2}$ and $u_{3}$ become aware that their packets have not been successfully received.*

### 2.2. Threshold Definition and Notation

Throughout the paper we define a load threshold $G^{\circ }$ as the maximum load such that the packet loss rate falls below a given target value $P_{L}^{\circ }$. In other words, when the instantaneous load *G* is below $G^{\circ }$, we have $P_{L}\leq P_{L}^{\circ }$, otherwise we have $P_{L}>P_{L}^{\circ }$.

In Table 1, some examples of probability distributions Λ(x) are shown with the corresponding target load threshold values. The first two rows in the table represent CRDSA schemes, where each user transmits the same number of replicas. The last two rows represent IRSA schemes, where the number of replicas per user is irregular. The values of $G^{\circ }$ have been obtained via a Monte Carlo simulation, for MAC frame length m=200 and target packet loss rate $P_{L}^{\circ }=0.01$.

**Figure 3:**
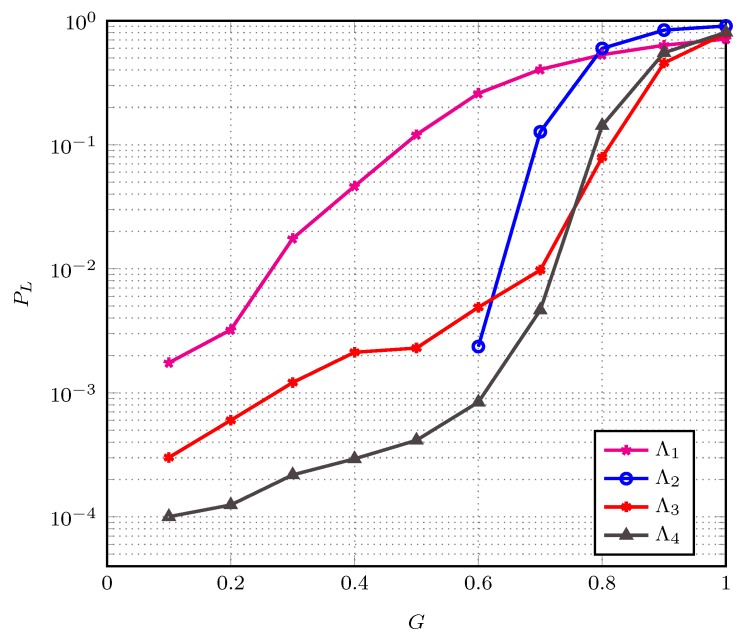
Packet loss rate $P_{L}$ versus instantaneous load *G* for frame length m=200 and the distributions in Table 1.

Figure 3 shows the packet loss rate $P_{L}$ versus instantaneous load *G* for the distributions in Table 1 and frame length m=200. As previously remarked, the SIC process in IRSA can be described by a bipartite graph, where unresolved collisions are associated with graphical structures known, in the low-density parity-check (LDPC) coding jargon, as stopping sets. It is well known that the impact of small stopping sets on the finite-length performance is strictly related to the fraction of degree-2 variable nodes in its bipartite graph and a similar role is played by degree-2 users in IRSA. As observed in the figure, the limitation of degree-2 repetition has a better error floor performance, but a poorer waterfall performance. The detailed packet loss rate performance analysis for IRSA schemes have been addressed in [5].

### 2.3. Combinatorial Parameters

We denote by $|\vec{v}|=\sum_{i=1}^{n}\left|v_{i}\right|$ the $\ell_{1}$ norm of a real-valued vector $\vec{v}=\left(v_{1},\cdots ,v_{n}\right)$. Moreover, given a second vector $\vec{w}=\left(w_{1},\cdots ,w_{n}\right)$ whose elements are nonnegative integers, we use the compact notation ${\vec{v}}^{\vec{w}}$ for $v_{1}^{w_{1}}\cdots v_{n}^{w_{n}}$.

Let $\vec{o}=\left(o_{1},\cdots ,o_{d_{\max}}\right)$ be a vector whose elements are all nonnegative integers. Let $M(\vec{o},b)$ be the set of all $|\vec{o}|\times b$ binary matrices M, with rows and columns indexed from 1 to $|\vec{o}|$ and from 1 to *b*, respectively, that fulfill the following properties: 1. The matrix M has the structure:

$$\begin{array}{c} M={\left[M_{1}^{T}\;\;M_{2}^{T}\;\;\cdots \;\;M_{d_{\max}}^{T}\right]}^{T} \end{array}$$

where $M_{i}$ has dimension $o_{i}\times b$ and all of its rows have Hamming weight *i*. 2. Every column of M has Hamming weight at least 2.

**Example** **2.** *Let $\vec{o}=\left(o_{1},o_{2},o_{3}\right)=\left(0,1,3\right)$ and b=5. Each matrix in $M\in M(\vec{o},b)$ has dimension 4×5. Its row indexes should be thought as partitioned into the two subsets {1} and {2,3,4}. The row of index 1 has weight 2, and the rows of indexes 2, 3, and 4 have weight 3. Every column of M has weight of at least 2. An example of matrix $M\in M(\vec{o},b)$ is:*

$$\begin{array}{c} M=\left(\begin{array}{ccccc} 1 & 0 & 0 & 1 & 0\\ 0 & 1 & 0 & 1 & 1\\ 1 & 1 & 1 & 0 & 0\\ 0 & 0 & 1 & 1 & 1 \end{array}\right)\;. \end{array}$$

The following lemma provides a formal expression for the cardinality of the set $M(\vec{o},b)$.

**Lemma** **1.** *For given $\vec{o}$ and b, let $h(\vec{o},b)$ be the cardinality of the set $M(\vec{o},b)$. Moreover, let $\vec{x}=\left(x_{1},x_{2},\cdots ,x_{|\vec{o}|}\right)$ and:*

(6) $$\begin{array}{c} \vec{q}=\left(\underset{o_{1}}{\underbrace{1,\cdots ,1}},\underset{o_{2}}{\underbrace{2,\cdots ,2}},\cdots ,\underset{o_{d_{\max}}}{\underbrace{d_{\max},\cdots ,d_{\max}}}\right). \end{array}$$

*Define the multivariate polynomials $A(\vec{x})$ and $B_{j,l}\left(\vec{x}\right)$ as:*

(7) $$\begin{array}{c} A\left(\vec{x}\right)=\prod_{i=1}^{|\vec{o}|}\left(1+x_{i}\right)-\left(1+\sum_{i=1}^{|\vec{o}|}x_{i}\right) \end{array}$$

*and:*

(8) $$\begin{array}{c} B_{j,l}\left(\vec{x}\right)={\left(\sum_{i=1}^{|\vec{o}|}x_{i}\right)}^{l}{\left(\prod_{i=1}^{|\vec{o}|}\left(1+x_{i}\right)\right)}^{j}. \end{array}$$

*Then, we have:*

(9) $$h(\vec{o},b)=\mathrm{coeff}({\left(A\left(\vec{x}\right)\right)}^{b},{\vec{x}}^{\;\vec{q}})$$

(10) =∑j=0b∑l=0b−jbjb−jl(−1)b−jcoeff(Bj,l(x→),x→q→)

*where $\mathrm{coeff}(P\left(\vec{x}\right),{\vec{x}}^{\;\vec{r}})$ is the coefficient of ${\vec{x}}^{\;\vec{r}}$ in the multivariate polynomial $P(\vec{x})$.*

**Proof**  Let ${\vec{c}}^{\;T}={\left(c_{1},\cdots ,c_{|\vec{o}|}\right)}^{T}$ be the generic column and define a multivariate enumerating function for valid columns (i.e., columns with weight of at least 2):

(11) $$\begin{array}{cc} A(\vec{x}) & =\sum_{\vec{c}:\left|\vec{c}\right|\geq 2}{\vec{x}}^{\;\vec{c}}. \end{array}$$

It is easy to recognize that an equivalent expression for $A(\vec{x})$ is the one shown in Equation (7). This is because $\left(1+x_{1}\right)\cdots \left(1+x_{|\vec{o}|}\right)$ provides the sum of all monomials in the variables $x_{1},\cdots x_{|\vec{o}|}$ with a unitary coefficient, to which we subtract all monomials of degrees 0 and 1 as required by the condition of validity.

Considering now *b* columns and applying properties of generating functions, $\mathrm{coeff}({\left(A\left(\vec{x}\right)\right)}^{b},{\vec{x}}^{\;\vec{w}})$ is the number of $|\vec{o}|\times b$ binary matrices such that all matrix columns are valid and such that the weight of row *i* is $w_{i}$. This immediately leads to Equation (9). The equivalent expression of Equation (10) is obtained by simple algebraic manipulation of the multivariate polynomial ${\left(A\left(\vec{x}\right)\right)}^{b}$. In particular, it is obtained by applying Newton’s binomial formula twice and by exploiting the identity $\mathrm{coeff}\left(\sum_{i}\alpha_{i}P_{i}\left(\vec{x}\right),{\vec{x}}^{\;\vec{w}}\right)=\sum_{i}\alpha_{i}\mathrm{coeff}\left(P_{i}\left(\vec{x}\right),{\vec{x}}^{\;\vec{w}}\right)$. □

## 3. Dynamic Access Probability Algorithm

In this section we introduce the proposed multiple access strategy based on a dynamic adjustment of the users access probability. Section 3.1 addresses estimation of the number of transmitting users; Section 3.2 exploits the developed estimator to perform congestion detection and resolution via dynamic access probability adjustment.

### 3.1. Number of Transmitting Users Estimation

In this subsection, we exploit frame configuration information at the end of SIC to estimate the number $t_{a}^{\left(k\right)}$ of transmitting users in the *k*-th frame, when an SIC failure occurs. For the sake of notational simplicity, the superscript (k) is temporarily omitted.

The total number of transmitting users is denoted by $t_{a}$. We also denote by $t_{a,l}$ the number of such users that employ the replica factor *l*. Clearly, we have $t_{a}=\sum_{l=1}^{d_{\max}}t_{a,l}$. The vector ${\vec{t}}_{a}=\left(t_{a,1},t_{a,2},\cdots ,t_{a,d_{\max}}\right)$ is referred to as transmitting users configuration at the beginning of the frame. The number of transmitted users that are recovered at the end of the SIC process is denoted by $\delta \leq t_{a}$. Out of these δ recovered users, $\delta_{l}\leq t_{a,l}$ are the ones using replica factor *l*, so that $\delta =\sum_{l=1}^{d_{\max}}\delta_{l}$. The vector $\vec{\delta }=\left(\delta_{1},\delta_{2},\cdots ,\delta_{d_{\max}}\right)$ is referred to as the recovered users configuration at the end of SIC.

Hereafter we develop a compact expression for the a posteriori probability distribution of the configuration ${\vec{t}}_{a}$ of transmitting users, given the number $m_{c}$ of residual collision slots and the configuration $\vec{\delta }$ of recovered users observed at the end of SIC. This probability is denoted by $P({\vec{t}}_{a}|m_{c},\vec{\delta })$. Note that, as transmitting users pick their slots uniformly at random, it is sufficient to condition to the number of collision slots (and not to their positions in the frame). The corresponding probability distribution of the number $t_{a}$ of transmitting users is given by:

(12) $$\begin{array}{c} P\left(t_{a}|m_{c},\vec{\delta }\right)=\sum_{{\vec{t}}_{a}:\left|{\vec{t}}_{a}\right|=t_{a}}P\left(\vec{t_{a}}|m_{c},\vec{\delta }\right). \end{array}$$

A maximum a posteriori (MAP) estimator for the number of transmitting users then returns the value:

(13) $$\begin{array}{c} {\hat{t}}_{a}=\underset{t_{a}}{\mathrm{argmax}}P\left(t_{a}|m_{c},\vec{\delta }\right). \end{array}$$

**Theorem** **1.** *The a posteriori probability distribution of the configuration ${\vec{t}}_{a}$ of the transmitting users fulfills:*

(14) P(t→a|mc,δ→)∝t→aδ→h(t→a−δ→,mc)∏l=1dmaxmlta,lP(t→a)

*where $h(\vec{o},b)$ is given by Lemma 1, t→aδ→=∏lta,lδl, and $P(\vec{t_{a}})$ is the a priori probability that the transmitting users configuration equals ${\vec{t}}_{a}$.*

**Proof.**  From Bayes’ rule we have:

(15) $$\begin{array}{cc} P(\vec{t_{a}}|m_{c},\vec{\delta }) & =\frac{P\left(m_{c},\vec{\delta }|\vec{t_{a}}\right)P\left(\vec{t_{a}}\right)}{P(m_{c},\vec{\delta })}\\ \; & \propto P\left(m_{c},\vec{\delta }|\vec{t_{a}}\right)P\left(\vec{t_{a}}\right). \end{array}$$

Let $T(\vec{t_{a}},m_{c},\vec{\delta })$ be the number of ways in which $|{\vec{t}}_{a}|$ transmitting users with configuration ${\vec{t}}_{a}$ can transmit their packet replicas in the frame so that, at the end of SIC, there are $m_{c}$ unresolved collision slots and a recovered users configuration $\vec{\delta }$. Moreover, let $T(\vec{t_{a}})$ be the number of ways in which $|\vec{t_{a}}|$ transmitting users with configuration ${\vec{t}}_{a}$ can place their packet replicas in the frame. The conditional probability $P(m_{c},\vec{\delta }|\vec{t_{a}})$ can be expressed as:

(16) $$\begin{array}{c} P\left(m_{c},\vec{\delta }|\vec{t_{a}}\right)=\frac{T(\vec{t_{a}},m_{c},\vec{\delta })}{T(\vec{t_{a}})}. \end{array}$$

The quantity $T(\vec{t_{a}})$ is readility shown to be given by:

(17) T(ta→)=∏l=1dmaxmlta,l.

To develop an expression for $T(\vec{t_{a}},m_{c},\vec{\delta })$, we proceed as follows. At the end of SIC, $|\vec{t_{a}}-\vec{\delta }|$ transmitting users with configuration $\vec{t_{a}}-\vec{\delta }$ remain unrecovered. The number of ways in which these users transmit their packet replicas to $m_{c}$ slots, forming $m_{c}$ collisions (at least two replicas per slot) is $h({\vec{t}}_{a}-\vec{\delta },m_{c})$. If we let $g(\vec{\delta },m_{c})$ be the number of ways in which $|\vec{\delta }|$ transmitting users with configuration $\vec{\delta }$ can place their packet replicas in a frame with $m-m_{c}$ free slots and $m_{c}$ unresolvable collision slots, so that SIC can recover all of them, we can write (no formal expression for $g(\vec{\delta },m_{c})$ is provided because this parameter, not depending on ${\vec{t}}_{a}$ does not play any role in the estimation process of Equation(13)):

(18) T(ta→,mc,δ→)=ta→δ→mmch(t→a−δ→,mc)g(δ→,mc).

Incorporating Equation (17) and Equation (18) into Equation (16) and then Equation (16) into Equation (15), and omitting all terms not depending on $\vec{t_{a}}$, we obtain Equation (14). □

Although Equations (13) and (14) define an exact MAP estimator, computing $h(\vec{t_{a}}-\vec{\delta },m_{c})$ turns out to be a complex task, becoming already intractable for frame sizes in the order of a few tens. For this reason we employ an approximated MAP estimator. In the approximation, all packet replicas, even from the same user, are regarded as distinguishable packets. Equivalently, each user chooses *l* slots with replacement. In this approximate setting, we have

(19) P(t→a|mc,δ→)∝t→aδ→h((∑l=1dmax(tl−δl)l),mc)m∑l=1dmaxtllP(t→a)

where $\left(\sum_{l=1}^{d_{\max}}\left(t_{l}-\delta_{l}\right)l\right)$ represents a vector with only one element, corresponding to $\vec{o}=\left(o_{1}\right)$ in $h(\vec{o},b)$. The value of $h(\left(\sum_{l=1}^{d_{\max}}\left(t_{l}-\delta_{l}\right)l\right),m_{c})$ is the number of ways in which $\sum_{l=1}^{d_{\max}}\left(t_{l}-\delta_{l}\right)l$ packet replicas are sent to $m_{c}$ slots, such that each slot receives not less than 2 packet replicas [25]; $m^{\sum_{l=1}^{d_{\max}}t_{l}l}$ is the total number of ways in which $\sum_{l=1}^{d_{\max}}t_{l}l$ packet replicas can be accommodated into the *m* slots.

As an estimator performance measure we consider the MSE, defined as:

(20) $$M_{\epsilon }=E\left[\epsilon^{2}\right]$$

where $\epsilon ={\hat{t}}_{a}^{\left(k\right)}-t_{a}^{\left(k\right)}$ is the estimation error.

### 3.2. Access Probability Adjustment Strategy

We say that we have a congestion on frame *k* whenever

(21) $$n_{a}^{\left(k\right)}>G^{\circ }m,$$

where we recall that $n_{a}^{\left(k\right)}$ is the number users that are active on frame *k*. Our purpose is to exploit the developed estimator to detect congestion states and dynamically adjust the users access probability to improve overall efficiency. Congestion states are resolved by tuning the access probability to control the number of transmitting users in the next frame.

The proposed scheme is based on the definition of three possible states for a frame, namely:

- **Not fully reliable estimate**. In high load conditions, SIC typically stops prematurely with a relatively small number of recovered users. We say that the estimate ${\hat{t}}_{a}^{\left(k\right)}$ is not fully reliable when the number of users recovered by processing the frame is smaller than the number of users that could not be recovered:
  (22) $$\delta^{\left(k\right)}<t_{a}^{\left(k\right)}-\delta^{\left(k\right)}$$
  or, equivalently,
  (23) $$\delta^{\left(k\right)}<t_{a}^{\left(k\right)}/2.$$
- **Congestion with reliable estimate**. The number of active users is above threshold $G^{\circ }m$, but the number of users recovered by processing the frame is not less than the number of users that could not be recovered:
  (24) $$n_{a}^{\left(k\right)}>G^{\circ }m\;\mathrm{and}\;\delta^{\left(k\right)}\geq t_{a}^{\left(k\right)}-\delta^{\left(k\right)}.$$
- **No congestion**. The number of active users is below threshold $G^{\circ }m$:
  (25) $$n_{a}^{\left(k\right)}\leq G^{\circ }m.$$

In the first case, a large number of transmitting users is unrecovered, and the packet loss rate is larger than 0.5. As illustrated in the numerical results section, the estimation MSE increases with the number of transmitting users and the estimate is therefore regarded as not suitable to design the access probability $p_{ac}$ in the subsequent frame. In contrast, in the last two cases the access probability in the next frame is calculated directly by employing the estimate of the number of transmitting users.

In the generic frame *k*, after all transmitting users have performed the transmission of their packet replicas, the receiver performs the SIC procedure. At the end of SIC, the receiver executes the procedure described in Algorithm 1. This procedure is executed regardless of the SIC termination status (success or failure). An explanation of Algorithm 1 is provided in the following.

**Algorithm 1:** Receiver procedure

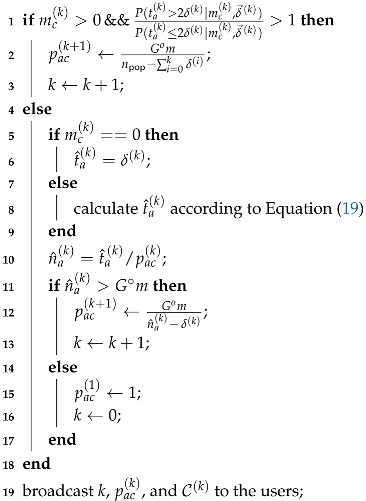



The first step (line 1) consists of detecting whether Equation (23) is fulfilled or not. When SIC succeeds ($m_{c}^{\left(k\right)}=0$), the estimation is perfect. The algorithm jumps to line 6 and simply sets ${\hat{t}}_{a}^{\;(k)}=\delta^{\left(k\right)}$. In case of an SIC failure ($m_{c}^{\left(k\right)}>0$), the algorithm applies a two-hypotheses MAP detector, whose development is presented in Appendix A, to decide whether Equation (23) holds (in which case estimation is considered unreliable) or not. Concretely, if

(26) $$\frac{P(t_{a}^{\left(k\right)}>2\delta^{\left(k\right)}|m_{c}^{\left(k\right)},{\vec{\delta }}^{\;(k)})}{P(t_{a}^{\left(k\right)}\leq 2\delta^{\left(k\right)}|m_{c}^{\left(k\right)},{\vec{\delta }}^{\;(k)})}>1,$$

then Equation (23) is assumed to hold and the estimation Equation (19) is regarded as not reliable enough. Otherwise, the estimate ${\hat{t}}_{a}^{\left(k\right)}$ is employed to design the access probability in the next frame.

When Equation (26) is satisfied, a ’not fully reliable estimate’ state is detected and the number of transmitting users is detected to be large enough to create a congestion but the relatively large estimation MSE prevents from relying on ${\hat{t}}_{a}^{\;(k)}$ to reliably adjust the access probability in the next frame. At the end of frame *k* a number $\sum_{i=0}^{k}\delta^{\left(i\right)}$ of active users have been recovered since the beginning of congestion. Therefore, at the beginning of the subsequent frame, the number of unresolved active users fulfills $n_{a}^{\left(k+1\right)}\leq n_{\mathrm{pop}}-\sum_{i=0}^{k}\delta^{\left(i\right)}$. To make the expected number of transmitting users in the subsequent frame below the target number $G^{\circ }m$, we set the access probability according to (line 2)

(27) $$\begin{array}{c} p_{ac}^{\left(k+1\right)}=\frac{G^{\circ }m}{n_{\mathrm{pop}}-\sum_{i=0}^{k}\delta^{\left(i\right)}}. \end{array}$$

This way, the conditional expected number of transmitting users in the next frame is

(28) $$\begin{array}{cc} E[T_{a}^{\left(k+1\right)}|n_{a}^{\left(k+1\right)}] & =n_{a}^{\left(k+1\right)}p_{ac}^{\left(k+1\right)}\\ \; & =\frac{n_{a}^{\left(0\right)}-\sum_{i=0}^{k}\delta^{\left(i\right)}}{n_{\mathrm{pop}}-\sum_{i=0}^{k}\delta^{\left(i\right)}}G^{\circ }m, \end{array}$$

where $n_{a}^{\left(0\right)}-\sum_{i=1}^{k}\delta^{\left(i\right)}$ represents the actual number of unrecovered active users at the beginning of frame k+1.

When the estimation result is detected to be reliable, an acceptable estimation MSE is assumed by the receiver, which exploits ${\hat{t}}_{a}^{\left(k\right)}$ (equal to $\delta^{\left(k\right)}$ in case of a SIC success or by Equation (19) in case of a failure) to obtain an estimate of the number of active users on frame *k*. Specifically, the receiver performs (line 10):

(29) $${\hat{n}}_{a}^{\left(k\right)}={\hat{t}}_{a}^{\left(k\right)}/p_{ac}^{\left(k\right)}.$$

The estimate ${\hat{n}}_{a}^{\left(k\right)}$ is compared with the threshold $G^{\circ }m$ (line 11). If ${\hat{n}}_{a}^{\left(k\right)}>G^{\circ }m$ the receiver declares a congestion with a reliable estimate state. The system is suffering from congestion, but most of (or all of) transmitting users have been recovered by SIC. The relatively low estimation MSE allows confidently using ${\hat{n}}_{a}^{\left(k\right)}$ to set the access probability for the next frame. If the access probability is kept unchanged in the subsequent frames, the number of transmitting users will deviate progressively from the target $G^{\circ }m$, leading to a low throughput. To make efficient use of channel resources, we increase the access probability in such a way as to maintain the number of transmitting users close to the target $G^{\circ }m$ in the next frame. From Equation (1), the estimated number of unrecovered active users at the beginning of the frame k+1 is ${\hat{n}}_{a}^{\left(k+1\right)}={\hat{n}}_{a}^{\left(k\right)}-\delta^{\left(k\right)}$. The target conditional expected number of transmitting users in frame k+1 is

(30) $$\begin{array}{c} E\left[T_{a}^{\left(k+1\right)}|n_{a}^{\left(k+1\right)}\right]=G^{\circ }m. \end{array}$$

Thus, the access probability over frame k+1 is set to (line 12):

(31) $$\begin{array}{c} p_{ac}^{\left(k+1\right)}=\frac{G^{\circ }m}{{\hat{n}}_{a}^{\left(k\right)}-\delta^{\left(k\right)}}. \end{array}$$

If ${\hat{n}}_{a}^{\;(k)}<G^{\circ }m$, a no congestion state is detected. The frame index *k* is re-initialized to 0 and the users access probability is set to be 1 (lines 15 and 16).

As a last step (line 19), the receiver broadcasts to the users the index of the next frame (index of the current frame increased by 1 if a congestion is detected and 0 otherwise), the access probability to be employed by active users in the next frame, and the list of indexes of collision slots at the end of SIC in the current frame. Upon receiving feedback from the receiver, users behave as follows:

- If k>0 (congestion), in the next frame each backlogged user attempts access to the frame with probability equal to the new access probability. Each non-backlogged user is prevented from transmitting new packets;
- If k=0 (no congestion), users that are in a backlog state retransmit their packet. Users that are not backlogged take their normal access activity.

Users are updated by the receiver about congestion or no congestion simply through the index *k*. Moreover, each of them knows whether or not it is in a backlog state simply by looking at the list of collision slot indexes $C^{\left(k\right)}$. Note that, if k=0 (no congestion) is broadcasted by the receiver, this does not necessarily mean that SIC has succeeded as there may be a small number of users unrecovered even though the system is not suffering from congestion. In this case, we simply let backlogged users retransmit packet replicas with probability 1 in the subsequent frame, together with possible fresh replicas from newly activated users.

## 4. Numerical Results

This section is organized into two subsections. In Section 4.1 we show results on the estimation of the number of transmitting users, while in Section 4.2 we address the performance achieved by the proposed scheme.

### 4.1. Estimation of Transmitting Users

In this section, we present Monte Carlo simulation results using the approximated estimator discussed at the end of Section 3.1. Let the frame length be m=200 and the user population size be $n_{\mathrm{pop}}=400$. Users are assumed to activate independently of each other at the beginning of every new frame. In each run, after users transmissions, SIC is applied at the receiver and then the developed approximated estimator is applied.

**Figure 4:**
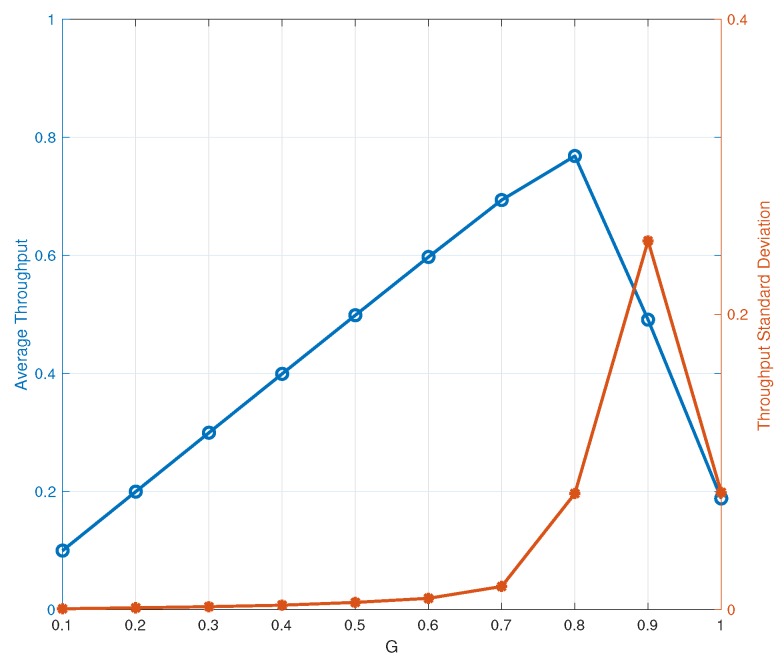
Average throughout and throughput standard deviation versus the instantaneous load *G* for IRSA with $\Lambda \left(x\right)=0.5x^{2}+0.28x^{3}+0.22x^{8}$.

Figure 4 shows the average throughput and throughput standard deviation versus the instantaneous load *G* for IRSA with $\Lambda \left(x\right)=0.5x^{2}+0.28x^{3}+0.22x^{8}$ [5]. The maximum average throughput is achieved at a value of *G* that is approximately equal to 0.8. However, the realizations of the per-frame throughput fluctuate around its statistical mean, the throughput standard deviation representing a reliable measure of the bobbing range (i.e., dispersion). A large standard deviation makes the average throughput a not fully meaningful parameter since, due to the per-frame throughput fluctuations, we have a higher probability that the system falls into a not fully reliable estimate state. In this respect, the peak average throughput is not necessarily a good working point, as the statistical mean alone is not able to capture the probability of falling into such an “outage” state.

**Figure 5:**
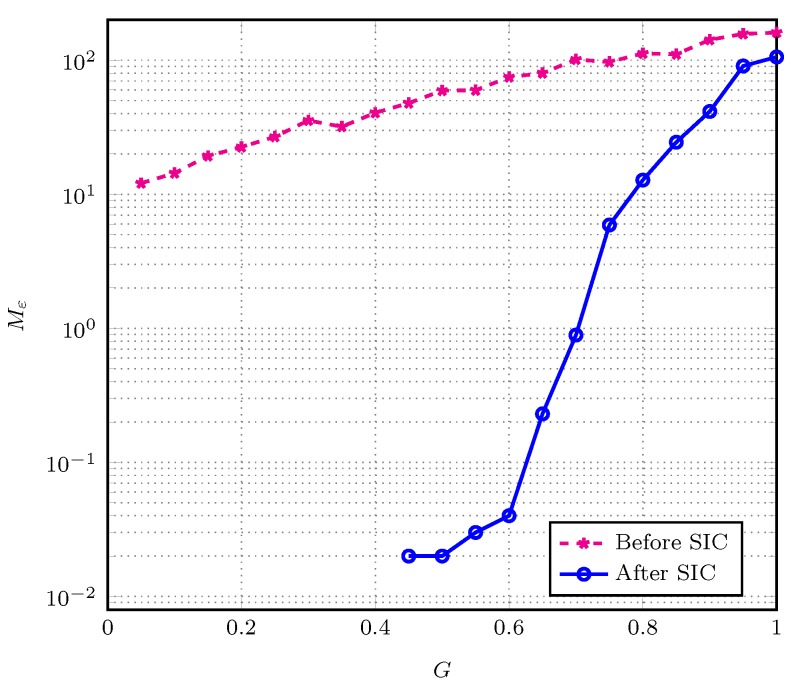
Mean squared error (MSE) versus *G* for IRSA with $\Lambda \left(x\right)=0.5x^{2}+0.28x^{3}+0.22x^{8}$.

Figure 5 shows the estimation performance after SIC iterations, letting the SIC-based receiver run until no active user is recovered. As a comparison, we also consider the estimation performance using the frame configuration before SIC iterations, which is reviewed in Appendix B. In the figure, the solid line is relevant to the proposed estimation making use of the frame configuration and recovered users configuration after SIC iterations. Moreover, a dashed line corresponds to the estimation based on the initially received frame, before SIC is applied. As observed in the figure, the proposed estimation algorithm is able to reduce the MSE effectively over the whole range of *G* values. It is also worth noting that the performance of the proposed estimator relies on the SIC performance. In low load conditions, the SIC procedure stops with a large number of users recovered, so in this region the proposed estimation algorithm is more effective. In contrast, in high load conditions SIC almost always stops prematurely, recovering a small number of users, leading the proposed estimation algorithm to be less effectively.

### 4.2. Dynamic Access Probability Simulation Results

In this subsection, we present the simulation results for dynamic access probability based coded random access schemes using the mentioned estimation methods. The frame length is m=200 and the user population size is $n_{\mathrm{pop}}=2000$. Moreover, the considered IRSA distribution is $\Lambda \left(x\right)=0.5x^{2}+0.28x^{3}+0.22x^{8}$. Each non-backlogged user activates, independently of the other users, with probability π=0.8 at the beginning of every frame with k=0. At the first frame, there are no backlogged users. The target traffic threshold $G^{\circ }$ is set to 0.65, 0.705, 0.80, and 0.938 respectively, of which $G^{\circ }=0.708$ is associated with $P_{L}^{\circ }=0.01$ and $G^{\circ }=0.938$ is the asymptotic threshold of the considered IRSA distribution [5]. The initial access probability is $p_{ac}^{\left(0\right)}=1$. We analyzed the system performance, during congestion resolution periods, through numerical simulations. Every simulation consisted of a sufficiently large number of runs and, in each run, the simulation was stopped when the congestion was resolved.

As a benchmark, consider transmission without any dynamic access probability adjustment process. The expected number of active users (transmitting users) in the initial frame is 1600. The average repetition rate is 3.6, corresponding to an expected number of 6480 packet replicas transmitted over the 200 slots. At the receiver, we have a vanishing probability to find singleton slots capable of triggering the SIC process. Without dynamic access probability adjustment, the packet loss rate becomes very close to 1 and the throughput very close to 0, meaning that almost no users are recovered in the subsequent frames, making system congestion unresolvable.

**Figure 6:**
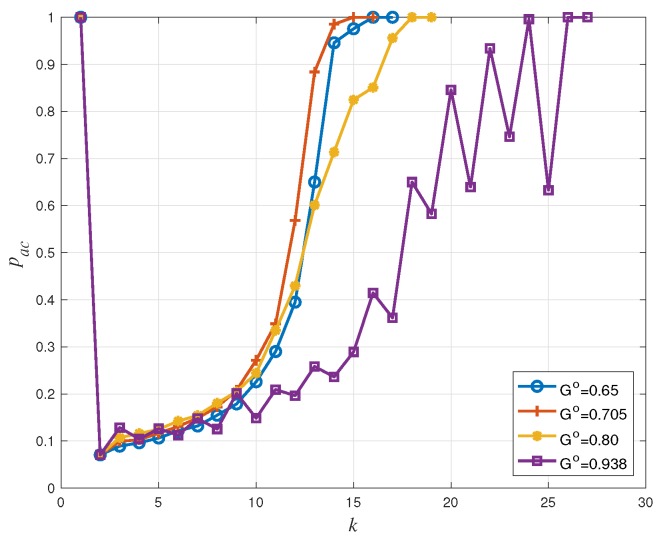
Access probability $p_{ac}$ versus frame index *k* for IRSA with $\Lambda \left(x\right)=0.5x^{2}+0.28x^{3}+0.22x^{8}$.

**Figure 7:**
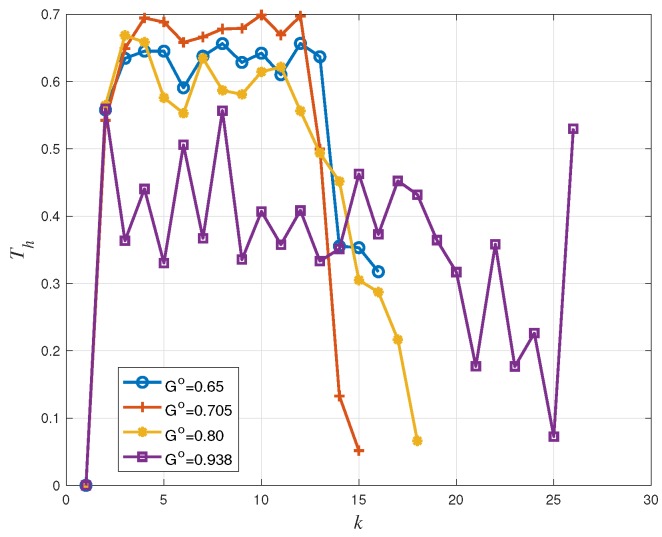
Throughput performance $T_{h}$ versus frame index *k* for IRSA with $\Lambda \left(x\right)=0.5x^{2}+0.28x^{3}+0.22x^{8}$.

**Figure 8:**
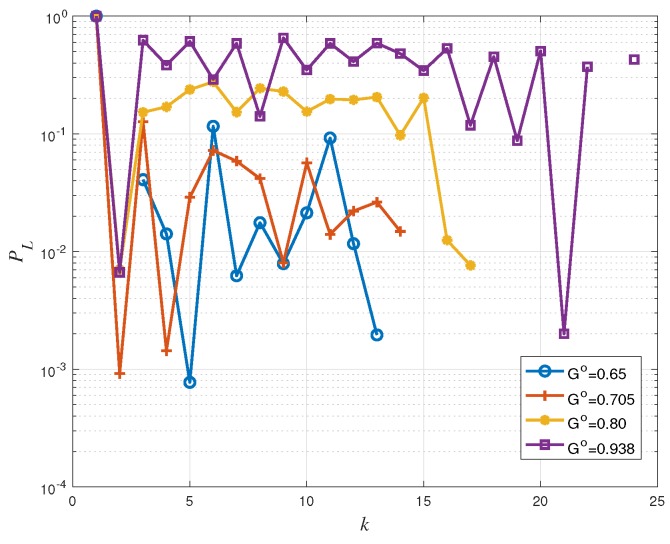
Packet loss rate $P_{L}$ versus frame index *k* for IRSA with $\Lambda \left(x\right)=0.5x^{2}+0.28x^{3}+0.22x^{8}$.

Figure 6, Figure 7 and Figure 8 show that the proposed access probability algorithm works well to resolve congestion. The users access probability is adjusted dynamically to track the number of active users. At frame 1, the access probability is decreased quickly to avoid working in the high load region. In this way, the estimator can provide a reliable estimate at the end of the frame and the receiver is able to perform an accurate access probability design for the users in the next frame. Then the access probability is adjusted dynamically to make the number of transmitting users around the target $G^{\circ }m$. It is increased slowly as some users are recovered by the receiver in each transmission. Each curve is plotted up to the maximum value of *k* for which congestions remain unresolved, which is different for the different choices of the target load threshold.

Back to Figure 3, we have seen that the IRSA scheme tends to show a packet loss rate floor at low offered traffic regimes, the floor appearing around $P_{L}=10^{-2}$ (corresponding to G=0.705) for $\Lambda \left(x\right)=0.5x^{2}+0.28x^{3}+0.22x^{8}$. For larger values of *G* (corresponding to the waterfall packet loss rate region), the packet loss rate increases rapidly: A $P_{L}≃0.08$ is achieved at G=0.80 and a $P_{L}≃0.5$ is achieved at G=0.938. Consequently, in Figure 8, systems with target $G^{\circ }=0.65$ and $G^{\circ }=0.705$ have a similar packet loss rate performance, and they perform better than those with target $G^{\circ }=0.80$ and $G^{\circ }=0.938$. Furthermore, due to estimation errors and to fluctuations of the actual number of transmitting users, we observe a minor packet loss rate deviation between Figure 8 and Figure 3. For example, in Figure 8, the packet loss rate with target $G^{\circ }=0.80$ is around 0.2, while in Figure 3, the packet loss rate at $G^{\circ }=0.80$ is approximately equal to 0.008.

As a final remark, recall that the throughput is defined as $G(1-P_{L})$. The influence of $P_{L}$ at G≤0.705 is small, so that the per-frame throughput $T_{h}^{\left(k\right)}$ is approximately equal to the instantaneous load *G*. That is why in Figure 7, the throughput performance with target $G^{\circ }=0.705$ is better than that with target $G^{\circ }=0.65$. However, for the cases $G^{\circ }=0.8$ and $G^{\circ }=0.938$, the influence of $P_{L}$ can not be ignored any more. The system performance is worse even though the load target $G^{\circ }$ is higher, since the packet loss rate is now considerably higher.

## 5. Conclusions

In this paper, we proposed a technique to estimate the number of transmitting users in each frame of an IRSA-based coded random access system. The estimated number of transmitting users in the current frame was exploited to adjust the users access probability in the next frame. Frame configuration information as well as recovered users configuration information at the end of the SIC procedure were employed to make the estimation more accurate. Numerical results revealed how the derived dynamic access probability strategy could resolve congestion efficiently, with a stable throughput and a target packet loss rate performance for a proper choice of the parameter $G^{\circ }$. Interesting directions of investigation include the exact efficient evaluation of the $h(\vec{o},b)$ function (addressed in Lemma 1), to make the optimum estimator applicable to large communication networks. Adjusting the frame length dynamically in situations of slowly varying traffic load over a large scale is another direction of investigation that, to the best of the authors’ knowledge, has not been so far addressed in the coded random access context.

## Figures and Tables

**Table 1:** Load threshold $G^{\circ }$ for different probability distributions Λ(x), for MAC frame length m=200, and packet loss rate target $P_{L}^{\circ }=0.01$.

Distribution, Λ(x)	$G^{\circ }$
$\Lambda_{1}\left(x\right)=x^{2}$	0.35
$\Lambda_{2}\left(x\right)=x^{4}$	0.69
$\Lambda_{3}\left(x\right)=0.5x^{2}+0.28x^{3}+0.22x^{8}$	0.705
$\Lambda_{4}\left(x\right)=0.25x^{2}+0.6x^{3}+0.15x^{8}$	0.76

## Appendix A. Justification and Implementation of Equation (uid44)

This appendix justifies and addresses the implementation of the two-hypotheses MAP detection rule Equation (26). Let the two hypotheses be $H_{0}$ and $H_{1}$. Moreover, let r represent the observation. The optimum detection rule consists of making the decision ${\hat{H}}_{0}$ when $P\left(H_{0}|r\right)>P\left(H_{1}|r\right)$ and of making the decision ${\hat{H}}_{1}$ otherwise.

In our case, $H_{0}$ corresponds to a ’not fully reliable estimate’ state (satisfied Equation (23)); $H_{1}$ corresponds to a ’reliable estimate’ state (Equation (23) not holding). The observation is $\left(m_{c}^{\left(k\right)},{\vec{\delta }}^{\;(k)}\right)$. Hence, we have:

(A1) $$\begin{array}{c} \frac{P(t_{a}^{\left(k\right)}>2\delta^{\left(k\right)}|m_{c}^{\left(k\right)},{\vec{\delta }}^{\;(k)})}{P(t_{a}^{\left(k\right)}\leq 2\delta^{\left(k\right)}|m_{c}^{\left(k\right)},{\vec{\delta }}^{\;(k)})}\overset{{\hat{H}}_{0}}{\underset{{\hat{H}}_{1}}{≷}}1, \end{array}$$

where $P\left(t_{a}^{\left(k\right)}>2\delta^{\left(k\right)}|m_{c}^{\left(k\right)},{\vec{\delta }}^{\;(k)}\right)=\sum_{t_{a}^{\;(k)}:\;t_{a}^{\;(k)}>2\delta^{\left(k\right)}}P\left(t_{a}^{\left(k\right)}|m_{c}^{\left(k\right)},{\vec{\delta }}^{\;(k)}\right)$ and $P\left(t_{a}^{\left(k\right)}\leq 2\delta^{\left(k\right)}|m_{c}^{\left(k\right)},{\vec{\delta }}^{\;(k)}\right)=\sum_{t_{a}^{\left(k\right)}:\;t_{a}^{\left(k\right)}\leq 2\delta^{\left(k\right)}}P\left(t_{a}^{\left(k\right)}|m_{c}^{\left(k\right)},{\vec{\delta }}^{\;(k)}\right).$ The probability $P(t_{a}^{\left(k\right)}|m_{c}^{\left(k\right)},{\vec{\delta }}^{\;(k)})$ can be expressed as:

(A2) $$\begin{array}{c} P\left(t_{a}^{\left(k\right)}|m_{c}^{\left(k\right)},{\vec{\delta }}^{\;(k)}\right)=\sum_{{\vec{t}}_{a}:\left|{\vec{t}}_{a}^{\;(k)}\right|=t_{a}^{\left(k\right)}}P\left({\vec{t}}_{a}^{\;(k)}|m_{c},{\vec{\delta }}^{\;(k)}\right), \end{array}$$

where $P({\vec{t}}_{a}^{\;(k)}|m_{c},{\vec{\delta }}^{\;(k)})$ comes from the estimator Equation (14).

## Appendix B. Estimation Using Collision Slots before SIC

The user’s repetition rate is Λ(x). Define $\Lambda^{\prime }\left(1\right)$ as the average user repetition rate given by $\Lambda^{\prime }\left(1\right)=\sum_{l}l\Lambda_{l}$. It is easy to verify that the probability that a generic user sends a packet replica within a given slot is $\Lambda^{\prime }\left(1\right)/m$. As the users send packet replicas randomly, the slot degree distribution is binomially distributed. The probability that a slot has *l* collided users is given by:

(A3) Ψl=talΛ′(1)ml1−Λ′(1)mta−l.

Before SIC iterations, the probability $p_{e}$ that a given slot is empty, the probability $p_{s}$ that a given slot is singleton and the probability $p_{c}$ that a given slot is a collision one can be expressed respectively as:

(A4) $$p_{e}={\left(1-\frac{\Lambda^{\prime }\left(1\right)}{m}\right)}^{t_{a}},$$

(A5) $$p_{s}=t_{a}\frac{\Lambda^{\prime }\left(1\right)}{m}{\left(1-\frac{\Lambda^{\prime }\left(1\right)}{m}\right)}^{t_{a}-1}$$

and:

(A6) $$p_{c}=1-p_{e}-p_{s}.$$

An estimation for $T_{a}$ using frame configuration before SIC performs:

(A7) $$\begin{array}{c} {\hat{t}}_{a}=\underset{t_{a}}{\mathrm{argmax}}P\;\;\left(t_{a}|w_{c}\right), \end{array}$$

where $w_{c}$ is the number of collision slots before SIC iterations. Following Bayes’ rule, $P(t_{a}|w_{c})$ may be developed as:

(A8) $$\begin{array}{cc} P(t_{a}|w_{c}) & \propto P\left(w_{c}|t_{a}\right)P\left(t_{a}\right)\\ \; & \propto {p_{c}}^{w_{c}}{\left(1-p_{c}\right)}^{m-w_{c}}. \end{array}$$
